# Adaptive Online Fault Diagnosis in Autonomous Robot Swarms

**DOI:** 10.3389/frobt.2018.00131

**Published:** 2018-11-30

**Authors:** James O'Keeffe, Danesh Tarapore, Alan G. Millard, Jon Timmis

**Affiliations:** ^1^Department of Electronic Engineering, University of York, York, United Kingdom; ^2^School of Electronics and Computer Science, University of Southampton, Southampton, United Kingdom

**Keywords:** swarm robotics, fault diagnosis, adaptive, autonomous, unsupervised learning

## Abstract

Previous work has shown that robot swarms are not always tolerant to the failure of individual robots, particularly those that have only partially failed and continue to contribute to collective behaviors. A case has been made for an active approach to fault tolerance in swarm robotic systems, whereby the swarm can identify and resolve faults that occur during operation. Existing approaches to active fault tolerance in swarms have so far omitted fault diagnosis, however we propose that diagnosis is a feature of active fault tolerance that is necessary if swarms are to obtain long-term autonomy. This paper presents a novel method for fault diagnosis that attempts to imitate some of the observed functions of natural immune system. The results of our simulated experiments show that our system is flexible, scalable, and improves swarm tolerance to various electro-mechanical faults in the cases examined.

## 1. Introduction

The synchronized operation of social insects, such as ants, is observed to be decentralized (Camazine et al., 2001). As a system, these insects exhibit behaviors that are robust, flexible and scalable (Şahin, 2004). These desirable features provide the motivation for research into swarm robotic systems.

Şahin (2004) proposed a criteria that swarm robotic systems should meet, namely that they should:

- Consist of autonomous robots
- Feature large numbers of robots
- Consist of homogeneous robots, or a few groups of homogeneous robots
- Consist of robots with only local sensing and communication abilities
- Consist of robots that are relatively incapable or inefficient with respect to the swarm or task at hand

Şahin (2004) identifies robustness, flexibility and scalability as properties that swarm robotic systems that fit this criteria should inherently possess. However, it was subsequently demonstrated that swarm robots are not always able to tolerate partially failed individuals (Winfield and Nembrini, 2006). It has also been shown that, where faults are present, the scalability of a system may also suffer (Bjerknes and Winfield, 2013). In light of this, Bjerknes and Winfield (2013) called for an active approach to improving fault tolerance, whereby the swarm is able autonomously to detect and resolve faults that occur in individual robots.

There are a number of links and algorithmic similarities between swarm systems and artificial immune systems (AIS), which are explored in detail by Timmis et al. (2010), who proposed that the similarities between swarm systems and AIS make them complimentary to one another. AIS are defined by De Castro and Timmis (2002) as “*adaptive systems, inspired by theoretical immunology and observed immune functions, principles and models, which are applied to problem solving.”* Bjerknes and Winfield (2013), reiterating the sentiment of Timmis et al. (2010), suggest that AIS may offer solutions to the fault tolerance problems they outline.

Cohen (2000) defines *maintenance* as the ability of the natural immune system to protect its host against the harm it will receive over the course of its life, comprising three stages:

1. Recognition—distinguishing what is normal from what is abnormal
2. Cognition—making decisions based on available information
3. Action—doing something as a result of any decisions made

The processes of recognition, cognition and action (RCA) could be considered analogous to fault detection, diagnosis, and recovery (FDDR) (Millard, 2016). If RCA can represent a natural immune response in biological systems then, by analogy, FDDR can represent an artificial immune response for engineered systems.

The existing literature on active fault tolerance in swarms examines fault detection alone (Tarapore et al., 2017) or fault detection and recovery (Khadidos et al., 2015), where recovery is only achieved by completely removing the faulty robot from the swarm. To the best of our knowledge, no research has yet been conducted with explicit regard to fault diagnosis in swarm robotic systems, as defined in Şahin (2004) (with the exception of our own previous work, O'Keeffe et al. (2017a) and O'Keeffe et al. (2017b). We propose that fault diagnosis is a necessary feature of active fault tolerance in order to ensure long-term autonomy. Operating in dangerous or inaccessible environments is given by Şahin (2004) as one of the motivations for swarm robotic research. In these environments, it will not always be possible to call on new robots to replace faulty ones. We propose that swarm robotic systems that are able to diagnose partially failed individuals and launch recovery actions (e.g., the replacement or repair of individual actuators or sensors in real-time) will be better suited to long-term autonomy in such environments.

In this work we present a novel fault diagnosis system that allows a simulated swarm robotic system to dynamically characterize and identify different types of faults in real time. We present our assessment of the system's scalability and flexibility to varying environments, as well as the results of sensitivity analysis to system noise and variable system parameters (found in Supplementary Material). The purpose of these experiments is to observe how varying parameter values affect system performance, and demonstrate the viability of our system for use with robot swarms. We then implement our diagnostic system on a simulated robot swarm performing the collective photo-taxis algorithm used in the work by Bjerknes and Winfield (2013), which provides the initial motivation for active fault tolerance in robot swarms. This work builds on our previous publication (O'Keeffe et al., 2017b) by presenting a mostly similar fault diagnosis mechanism with some modifications and subjecting it to more rigorous experimentation. The extensions made by this work are:

1. The introduction of the possibility that diagnostic tests *T*_1−6_ (detailed in section Fault Diagnosis) will classify a fault incorrectly.
2. The introduction of active memory allocation
3. Scalability and Flexibility Analysis (section Scalability and Flexibility)
4. Sensitivity analysis performed for noise (section Noise Sensitivity) and system parameters (see Supplementary Material)
5. Quantitative swarm performance assessment (section Collective Photo-Taxis)

This work aims to demonstrate and quantify how our proposed immune-inspired fault diagnosis mechanism addresses the problems on fault tolerant robot swarms that are discussed by Winfield and Nembrini (2006) and Bjerknes and Winfield (2013), as well as highlighting some of its limitations.

## 2. Related work

We approach this work with the long-term goal of producing fully integrated FDDR for a swarm robotic system. Within FDDR there are dependencies between the sub-processes. To illustrate, a fault must be detected before it can be diagnosed, recovery must be performed to establish the validity of a diagnosis autonomously and so on. The interrelation of processes within FDDR makes it a large problem to approach as a whole. For that reason, previous research into FDDR in swarm robotics typically only examines one process in significant detail.

### 2.1. Fault detection for robot swarms

Fault detection in robot swarms has been approached in a number of ways. Christensen et al. (2009) present a system that is inspired by the behavior of fireflies, whereby robots are each equipped with periodically flashing light-emitting diodes (LEDs) that they try to synchronize with each other. The swarm is able to detect faults when an individual robot fails to flash its LED at suitable intervals, and this is recognized by a neighbor in the swarm. Millard (2016), on the other hand, uses comparisons between real-time observations of a robot's behavior and its expected behavior (obtained via online simulations), in order to detect faults. In both of these studies, the fundamental mechanism that enables faults to be detected is, implicitly or explicitly, the comparison of an observed robot behavior with its expected behavior, where a discrepancy between the two indicates the presence of a fault. This comparison is common to all previous approaches to fault detection in swarm robotics.

The medium used for robot behavior comparisons varies depending on the system under consideration. For example, Khadidos et al. (2015) instruct each robot in a swarm to broadcast information from its sensors, position coordinates and motor values to its neighbors, and vice-versa. Based on this information, each robot can decide where it believes its neighbor should be relative to itself, by calculating the distance and angle between each other's coordinates. If a robot reports a state that is contradictory to the states reported by multiple neighboring robots, it is assessed to be faulty. Similarly, Tarapore et al. (2017) use behavioral feature vectors (BFVs) to encode a robot's behavior as a vector of Boolean features—where 1 and 0 indicate a present or absent behavioral feature, respectively. Tarapore et al. (2017) implement fault detection such that BFVs possessed by a majority of the robots in a local swarm are interpreted as normal, whilst BFVs that are possessed by only a minority of robots are indicative of a fault present in those robots. An advantage of the approach taken by Tarapore et al. (2017) is that BFVs can be used to detect faults without the need for an *a priori* model of normal behavior (although some *a priori* domain-specific knowledge is still required to define BFVs for a robot swarm).

### 2.2. Fault diagnosis for multi-robot systems

Proceeding fault detection, the next stage in FDDR is fault diagnosis. Where fault detection is a process that identifies deviations from normal behavior, fault diagnosis is a process that identifies the root cause of the deviation. There have been a number of approaches to fault diagnosis in multi-robot systems. We make the distinction between multi-robot systems and swarm systems here, using the definition given by Şahin (2004), either because of hierarchical control structures or consisting of too few robots without the suggestion of scalability. Although the fault diagnosis approaches discussed in this section are not explicitly designed for swarms, the described diagnostic techniques inform our work toward fault diagnosis in robot swarms.

Winfield and Nembrini (2006) demonstrated that different types of fault cause robot behavior to deviate from what is expected in different ways. As faults can be detected by observing the discrepancies between expected and observed robot states, faults can be diagnosed by examining what specific states or features are discrepant. For example, Daigle et al. (2007) use the discrepancies between model-predicted behavior and observed robot behaviors to create a “fault signature” for different types of faults, which can then be used to diagnose faults that occur in real-time. Similarly, Carrasco et al. (2011) model the behavior of normally functioning and faulty robots offline. Faults are detected based on discrepancies between the model-predicted normal robot state and measured robot states during operation. Faults are then isolated by which modeled state for faulty behavior most closely resembles the measured robot state. One limitation of such approaches is the lack of capability to learn or adapt to dynamic fault signatures, and rely on comprehensive prior modeling in order to be effective. To retain the advantages of BFVs, we would argue that it is more appropriate for an autonomous fault diagnosis mechanism to establish models of faulty behavior online, in which case the resulting system will bear a closer resemblance to Learning Classifier Systems (Shafi and Abbass, 2017) than the supervised learning methods described by Daigle et al. (2007) and Carrasco et al. (2011), and used in our earlier work O'Keeffe et al. (2017a). Faults can also be diagnosed through more explicit assessment. Kutzer et al. (2008) implement diagnosis such that faulty robots perform diagnostic maneuvres, consisting of various tests designed to isolate the root-cause of a fault. A trained probabilistic model is then used to estimate the state of the faulty robot based on its observed performance of the diagnostic maneuvres.

### 2.3. Bio-inspired artificial immunity for robot swarms

Previous research toward fault detection and fault diagnosis in robotic systems are obvious considerations for our work. However, our work toward fault diagnosis is conducted in the context of creating “artificial immunity” for robot swarms.

The vertebrate immune system is observed to be able to recover its host body from harm and illness with which its host was previously unfamiliar (Capra et al., 1999). Of equal importance is the natural immune system's ability to *remember* what it has previously learned, allowing it to recover from known illnesses in a more efficient manner (Capra et al., 1999).

When the vertebrate immune system encounters an infection, the lymphocytes that recognize pathogens proliferate and differentiate (Janeway, 1992). Once the pathogens have been destroyed, most of the immune cells that were involved in the immune response are eliminated, except for a small population of long-lasting elements made up of the immune cells which most effectively fought the pathogens (Floreano and Mattiussi, 2008), known as *memory* cells (Kindt et al., 2007). These cells have an increased sensitivity to antigens and will prompt a faster immune response should their host encounter the same pathogen again (Floreano and Mattiussi, 2008). As this process is invaluable in maintaining a host organism for a natural immune system, so too will it be for maintaining a robot swarm—an AIS should seek to minimize any down-time within a swarm as this is where the system will be most vulnerable to further faults. For example, for emergent behaviors requiring *n* functioning robots, the swarm system will be less vulnerable to faults, in terms of task performance, if it consists of *n*+1 functioning robots than the bare minimum *n*. AIS should not only be able to diagnose faults that are occurring for the first time, but also be able to characterize these faults for comparison against any other faults that might occur in the future. When subsequent faults do occur, the system should be able to recognize if that fault is similar to one it has previously encountered and diagnose it more efficiently.

We have argued that fault diagnosis necessitates an integrated fault detection mechanism. The suitability of BFVs for fault detection in robot swarms has been demonstrated by Tarapore et al. (2017). In our previous work (O'Keeffe et al., 2017a), we demonstrated that we could design BFVs for a swarm robotic system that could then be used to classify different types of fault using a decision tree Quinlan (1986).

The use of BFVs in FDDR relies on a BFV consisting of features that adequately represent normal robot behavior, as well as deviations from normal robot behavior. We achieve this by considering robot hardware, behavior(s) and the types of fault a given system could be vulnerable to.

## 3. Methods

We now propose a novel method for adaptive fault diagnosis, using BFVs as a medium for fault characterization. This system will be subjected to a series of experiments, detailed later in this section, to assess its viability for improving fault tolerance in robot swarms.

### 3.1. Experimental setup

We use simulated models of the marXbot (Bonani et al., 2010) swarm robotic platform. These are two-wheeled, 17*cm* diameter swarm robots, with on-board proximity and range and bearing (RAB) sensors. In the experiments conducted for this work the robots exhibit flocking, aggregation, obstacle avoidance and collective photo-taxis swarm behaviors. In all of these behaviors, each robot moves in response to the position of its neighbors and/or point of interest. In our experiments, a normally functioning robot will always be motional.

The faults types we examine are chosen based on a cross-section of the literature that informs our work. Therefore, some of the fault types are not necessarily chosen with explicit regard to the marXbot platform, but with respect to an as-yet undefined swarm robotic system that we conceive of performing in the circumstances described by Şahin (2004).

The faults considered in this work are:

- **Software hang (****H**_1_**)**: This fault will cause a robot to get stuck performing whatever action it was performing at the last moment it was normally functioning. Software hang is given by Christensen et al. (2008) as an example of a fault that necessitates exogenous approaches to fault detection.
- **Power failure (****H**_2_**)**: A robot that suffers a power failure will completely stop moving and remain unresponsive to its surroundings. Power failure is a recurring example in work on fault tolerance (Carlson, 2004; Winfield and Nembrini, 2006).
- **Complete Sensor failure (****H**_3_**)**: For a complete sensor failure an individual robot will be unable to detect the presence of any neighbors, light sources or obstacles. Sensor failure is another recurring example used in fault tolerant swarm research (Carlson, 2004; Winfield and Nembrini, 2006).
- **Complete Motor failure (****H**_4_**)**: A complete motor failure will cause one of the afflicted individual's motors to stop and henceforth become unresponsive to its controller. The other motor will remain functional, meaning that the afflicted robot will either stop completely if it is trying to turn using its faulty motor, or otherwise be limited to turning on the spot. In the study by Winfield and Nembrini (2006) motor failure is the most damaging to collective behavior, and therefore an obvious choice for inclusion in this study.
- **Partial Motor failure (****H**_5_**)**: For partial motor failure the individual's faulty motor will remain responsive, however it will only allow its associated wheel to turn at half speed. Another fault type included in the study by Winfield and Nembrini (2006), partial motor failure is less damaging to collective behavior than complete motor failure, but still results in a reduction in overall swarm performance.
- **Partial Sensor failure (****H**_6_**)**: For a partial sensor failure a robot will only be able to detect the presence of neighbors, light sources and obstacles within ±60 ° of its current heading. This is similar to the definition of partial sensor failure used by Millard (2016) in their work on fault tolerant robot swarms.

The robot BFV used in this work, where the binary vector *BFV*(*t*) = [*F*_1_(*t*), *F*_2_(*t*), *F*_3_(*t*), *F*_4_(*t*), *F*_5_(*t*), *F*_6_(*t*)], is designed by considering, in order, the robot's hardware, its behavior(s) and potential fault types—arriving at the following set of features:

(1) $$\begin{array}{l} F_{1}\left(t\right)=1\text{ if }N_{R}\left(t\right)>0,\text{otherwise }F_{1}\left(t\right)=0 \end{array}$$

where *N*_*R*_(*t*) is the total number of neighbors in sensing range (approximately 1*m*) of the robot at time *t*.

(2) $$\begin{array}{l} F_{2}\left(t\right)=1\text{ if }N_{C}\left(t\right)>0,\text{otherwise }F_{2}\left(t\right)=0 \end{array}$$

where *N*_*C*_(*t*) is the total number of neighbors at a distance less than the close proximity threshold, *C*, (approximately 0.3*m*) from the robot at time *t*.

(3) $$\begin{array}{l} F_{3}\left(t\right)=1\text{ if }|v\left(t\right)|>0.8|v_{max}|,\text{otherwise }F_{3}\left(t\right)=0 \end{array}$$

where |*v*(*t*)| is the magnitude of linear velocity at time *t* and |*v*_*max*_| is the maximum linear velocity a robot can achieve (approximately 5*cms*^−1^ in this work).

(4) $$\begin{array}{l} F_{4}\left(t\right)=1\text{ if }|v\left(t\right)|>0.2|v_{max}|,\text{otherwise }F_{4}\left(t\right)=0 \end{array}$$

(5) $$\begin{array}{l} F_{5}=1\text{ if }|\theta \left(t\right)|>0.4|\theta_{max}|,\text{otherwise }F_{5}\left(t\right)=0 \end{array}$$

where |θ(*t*)| is the magnitude of angular velocity at time *t* and |θ_*max*_| is the maximum angular velocity a robot can achieve (approximately 24°*s*^−1^ in this work).

(6) $$\begin{array}{l} F_{6}\left(t\right)=1\text{ if }H_{1}=True\text{ at time }t,\text{otherwise }F_{6}\left(t\right)=0 \end{array}$$

*F*_6_ is inspired by the *watchdog timer* (Huang and Selman, 1986) commonly found in embedded systems. If a robot's software becomes stuck or crashes, its feature *F*_6_ will return a value of 1.

Features are updated at each control-step with threshold values for *F*_3_, *F*_4_ and *F*_5_ set to 80% of *v*_*max*_ (Equation 3), 20% of *v*_*max*_ (Equation 4) and 40% of ω_*max*_ (Equation 5), respectively. These thresholds allow for clear distinctions in individual robot behavior to be represented in the robot's BFVs whilst also being tolerant of simulated electro-mechanical noise, as we demonstrated previously in O'Keeffe et al. (2017a).

Whilst the six features described are only one possible imagining of a BFV for our system, we demonstrated in O'Keeffe et al. (2017a) that *F*_1_−*F*_5_ were each individually discriminatory when a decision tree was used to classify the same six fault types, and the merits of *F*_6_ for classifying software hang faults were clearly demonstrated in our subsequent work (O'Keeffe et al., 2017b).

In their work on fault detection, Christensen et al. (2008) propose that purely endogenous approaches are inadequate, using the example of a robot suffering controller software hang being unable to communicate this to the swarm, and propose that exogenous approaches are necessary. However, based on knowledge of the system we are working with, purely exogenous approaches will also be inadequate—it would be very difficult in some cases for an independent observer to distinguish a robot afflicted with complete sensor failure, which has has caused it to get stuck against a wall, from a robot that happened to be near a wall when its motors failed, rendering it motionless, as both faults have the same effect on the robot's observed behavior. We propose that a combined endogenous and exogenous approach, whereby each robot estimates its own BFVs proprioceptively as well as exteroceptively estimating the BFVs of any neighbors in range, would be less susceptible to the problems outlined with either approach individually.

*F*_1−2_ are estimated proprioceptively by the robot's own RAB sensor, and exteroceptively by the RAB sensors of the robots neighbors (as the swarm is homogeneous, if a given robot can detect a neighbor, the neighbor can detect that robot). *F*_3−5_ are estimated proprioceptively by the robot monitoring its own controller output to motors, and exteroceptively by the rate of change in readings from a neighbor's RAB sensor.

### 3.2. Integrated fault diagnosis

Here we detail our proposed system for fault diagnosis and how it is integrated with provisional fault detection and recovery mechanisms for an autonomous approach to FDDR:

#### 3.2.1. Fault detection

Each of the faults described in section Experimental Setup will cause some discrepancy between proprioceptively estimated features, $F_{1-6}^{p}$, and exteroceptively estimated features, $F_{1-6}^{e}$. In our previous work, O'Keeffe et al. (2017b), we demonstrated that this could be used as a simple fault detection mechanism, as it ensures that faults are represented in the set of BFVs that are analyzed during diagnosis. Faults are detected according to Equation 7

(7) $$\begin{array}{l} \text{fault detected at time }t=true\text{ if}\overset{T}{\underset{t=T-W}{\sum }}d\left(t\right)\geq \rho W\text{ for }T>W \end{array}$$

Where *T* indicates the time elapsed since a robot started being observed by a neighbor. The binary value *d* is set according to Equation 8, where *d* = 0 if there is agreement between exteroceptively and proprioceptively estimated robot features. A robot will make *W* observations of its neighbor, once per control-step, at discrete times, *t*, before a fault can be detected, where *t* indicates the time elapsed since the start of an experiment. The value of ρ indicates what proportion of the *W* observations must prompt the value of *d* to be equal to 1 in order for a fault to be detected.

(8) $$\begin{array}{l} d\left(t\right)=\{\begin{array}{cc} 0\; & \text{if }F_{i}^{p}\left(t\right)=F_{i}^{e}\left(t\right)\text{for }i=1,..,6\\ 1\; & \text{otherwise} \end{array} \end{array}$$

This approach to BFV-based fault detection, whilst far simpler than that presented by Tarapore et al. (2017), shares the benefit of not needing an *a priori* model of faulty behavior.

When a fault is detected in a robot by another member of the swarm, the set of BFVs, comprising the faulty robot's proprioceptively estimated features and the detecting robot's exteroceptively estimated features for the faulty robot, which prompted the fault to be detected, can be used as the BFV signature for that particular fault.

#### 3.2.2. Fault diagnosis

BFVs are an effective medium for comparing fault signatures where there is a known reference for each type of fault. However, constructing *a priori* models of faulty robot behavior negates the advantages of BFV-based fault detection, as described by Tarapore et al. (2017). To retain these advantages, there needs to be a way of associating fault categories with their respective BFV signatures online.

As the types of fault that may occur in robots can be anticipated using techniques such as Failure Mode and Effect Analysis (FMEA) (Dailey, 2004) and Fault Tree Analysis (FTA) (Ericson and Ll, 1999), it is a realistic possibility to design a series of tests that can isolate the root cause of the fault. These tests are assessed in real-time by another robot in the swarm, in a similar fashion to the diagnostic maneuvres used by Kutzer et al. (2008), which declares itself as the assessing robot once a fault has been detected. These tests are designed in sympathy with a type of fault such that a robot will only be able to pass if it is not affected by that type of fault. A robot performs every test in sequence—a brute-force approach to diagnosis—until it passes them all or it fails one, which indicates the presence of an associated fault. In this manner, different categories of fault can be identified and thus associated with their BFV signatures in the first instance.

When an assessing robot initiates a diagnostic test routine, it approaches the faulty robot and instructs it to stop moving so that it can begin its assessment. Whilst it is performing its diagnostic routine, the assessing robot will also act as a beacon, instructing other approaching robots in the swarm to turn away so as not to interfere.

The diagnostic tests are arranged in order such that the faults which most severely inhibit robot behavior are tested for first, as some faults could obscure the test results for others. For example, a robot suffering power failure would also fail motor and sensor tests. The diagnostic tests we use, and the respective faults they test for, are as follows:

- **Software hang (*****T***_1_**)**: This occurs immediately after the assessing robot moves into testing range. In order for the faulty robot to perform later diagnostic tests, it must be able to communicate with the assessing robot. To establish this communication, the assessing robot first pings the faulty robot and instructs it to stop moving. If the faulty robot is suffering from software hang, it will disregard this request. If the faulty robot is persistently unreachable and ignores the assessing robot's stop request, it will be diagnosed as having software hang. This relies on the assumption that communication between normally operating robots is reliable.
- **Power failure (*****T***_2_**)**: Almost identical to *T*_1_, however in cases where the robot is suffering from power failure it will also stop moving. The assessing robot therefore diagnoses power failure in instances where the faulty robot is unreachable but stationary. It is acknowledged that this would only work for normal behaviors where robots should always be in motion. For a swarm exhibiting behavior that necessitated periods where the robot was stationary, the relationship between *T*_1_ and *T*_2_ would have to be revised.
- **Complete Sensor failure (*****T***_3_**)**: Once the faulty robot has been established as responsive to the assessing robot, the two robots should have settled within range of each other—something that will be reflected in their BFV. A complete sensor failure is diagnosed if the faulty robot's feature $F_{1}^{p}$ returns a value of 0.
- **Complete Motor failure (*****T***_4_**)**: The faulty robot is asked to spin on its right and left wheels. If the faulty robot receives and processes this request but is unable to perform one or both of the turns, it is decided that the robot is suffering from complete motor failure. This is ascertained by monitoring the faulty robot's feature $F_{4}^{e}$, where a returned value of 0 indicates the robot has failed the test.
- **Partial Motor failure (*****T***_5_**)**: Having established that both of its motors are responsive, the faulty robot is then asked to demonstrate that both motors work together as they should. The faulty robot is asked to move forward in a straight line. If the faulty robot is observed to move in an arc or otherwise deviate from moving in a straight line, it is decided that the faulty robot is suffering from partial motor failure. This is achieved by monitoring the faulty robot's feature $F_{5}^{e}$, where a returned value of 1 indicates that the robot has failed the test.
- **Partial Sensor failure (*****T***_6_**)**: To test for partial sensor failure, the faulty robot performs a 360° turn whilst acknowledging the presence of the assessing robot. If the faulty robot fails to acknowledge the assessing robot for any period of this turn, the robot is decided to be suffering from partial sensor failure.

The use of a finite number of pre-written diagnostic tests will only allow for the diagnosis of a finite number of distinct categories—something that could be considered inflexible. However, we counter that there is an equivalence between this approach and our understanding of the vertebrate immune system. The vertebrate immune system is reliant on an exceedingly large lymphocyte repertoire that has the ability to bind to an exceedingly large number of potential pathogens (Owen et al., 2013). This property of the natural immune system is the product of cross-generational evolution rather than something learned in the lifespan of a single host body (although the specific composition and distribution of the repertoire does change during host life span).

For a swarm robotic system with finite functionality, there will, at a high level, be a finite number of discrete fault types a robot can be affected by, such as actuator failure, sensor failure or power failure. Each of these faults can theoretically then be identified by a corresponding diagnostic test. We propose that the resulting repertoire of diagnostic tests that resolve these fault types can be considered analogous to the lymphocyte repertoires observed in the natural immune system—and the use of techniques like FMEA to identify a series of fault types analogous to natural evolutions spurring of the production of lymphocyte repertoires in individual host bodies (albeit an accelerated version). In the event that a fault occurred in a robot that did not fall into one of the identifiable categories, or for any reason was not recognizable as such, the effect would be the same as when the natural immune system is unable to recognize or remove harmful pathogens; the problem persists and, if the host is not tolerant, it eventually perishes.

#### 3.2.3. Fault recovery

When a fault is diagnosed, the system must be able to ascertain whether or not the diagnosis was appropriate. This is done by carrying out a corresponding recovery action and then subsequently observing the faulty robot to check whether the fault persists. We propose that all six fault types described in section Experimental Setup can be resolved by one of three recovery actions:

- **Power Cycle (*****R***_1_**)**: It is assumed for this work that, when a robot has its power cycled, it is turned back on with a new or replenished power source. This recovery action resolves software hang (*H*_1_) and power failure (*H*_2_) faults.
- **Sensor Replacement (*****R***_2_**)**: Replacement of robot sensors resolves complete and partial sensor failures (*H*_3_ and *H*_6_, respectively).
- **Motor Replacement (*****R***_3_**)**: Replacement of robot motors resolves complete and partial motor failures (*H*_4_ and *H*_5_, respectively).

We acknowledge that carrying out these proposed recovery actions autonomously with the marXbot platform is not realistic at the time of writing, and all recovery actions are simulated for this work. However, we anticipate that future swarm robotic platforms will possess the ability to perform similar actions and, as with the selection of faults, the diagnosis system presented here is designed with intention of being implemented on such a platform in the future.

When a recovery action is executed, the faulty robot continues to be observed by the assessing robot. If the faulty robot's exteroceptively and proprioceptively estimated BFVs match after a recovery action has been performed, the fault is considered to be resolved.

In the event that a robot's fault persists after having attempted diagnostic tests *T*_1−6_, the faulty robot is declared to be a lost cause and shut down. The faulty robot will now be, for all intents and purposes, an inanimate object in the arena.

If, on the other hand, a robot is subjected to diagnostic tests and passes them all, the faulty robot is treated as a false-positive and is allowed to continue operating normally. The fault signature is considered void in this case and not used for any subsequent comparison.

#### 3.2.4. Fault memory

Each time a fault is successfully resolved, the BFV signature for that fault and the respective diagnosis are stored in the assessing robot's memory, which is then shared with other members of the swarm via local communication. When subsequent faults occur, the BFV representations of those faults can be checked for similarity against the BFV representation of previous faults.

Similarity between two faults is established by finding the Pearson correlation coefficient (Benesty et al., 2009) between their BFV signatures (Equation 9):

(9) $$\begin{array}{l} r=\frac{\sum_{m}\sum_{n}\left(F_{mn}-\bar{F}\right)\left(F_{0_{mn}}-\bar{F_{0}}\right)}{\sqrt{{\left(\sum_{m}\sum_{n}F_{mn}-\bar{F}\right)}^{2}{\left(\sum_{m}\sum_{n}F_{0_{mn}}-\bar{F_{0}}\right)}^{2}}} \end{array}$$

Where *r* is the correlation coefficient between a current and previous fault signature, *F* and *F*_0_, respectively, where *F* and *F*_0_ are two dimensional binary data sets. $\bar{F}$ and $\bar{F_{0}}$ are the mean values of sets *F* and *F*_0_, respectively, and *m* and *n* indicate positional index within the 2D binary data set (the specific orientation of *m* and *n* is arbitrary for this equation).

When attempting to diagnose a fault from memory, the previously stored fault which produces the greatest *r*-value (Equation 9) is considered the most likely fault type and recovery is initiated in sympathy with that fault. The minimum *r*-value required for two faults to be considered similar, and thus eligible for this process, is investigated in this work.

The system we propose here is adaptive; we discussed in O'Keeffe et al. (2017a) and O'Keeffe et al. (2017b) how individual fault types could affect robots in different ways, according to the robot's circumstances. This is similar to the evolution of pathogens which makes it harder for the vertebrate immune system to detect and destroy them. In our system, as long as a fault is detectable and diagnosable by our defined tests, its BFV representation will be committed to memory. In this way our system's representation of each fault type is dynamic and can evolve in real time, as the vertebrate immune system is able to adapt to evolving pathogens (Floreano and Mattiussi, 2008). We would therefore argue that our system, at a high level, mirrors the desirable characteristics of the vertebrate immune system described in the previous section.

### 3.3. Experimental setup

All experiments for this work were conducted using Autonomous Robots Go Swarming (ARGoS) (Pinciroli et al., 2011), a physics based, discrete-time, multi-robot simulator that supports a variety of swarm robotics platforms exhibiting user-defined behaviors. The use of a simulator is temporary and intended as a proof-of-principle for the proposed system before beginning work with hardware platforms. We use simulated models of marXbots for all experiments, as we consider this a representative swarm robotic platform. Features are updated at every control-cycle (10*ms*).

For all experiments we apply Gaussian noise to the raw linear and angular velocity readings obtained by robot's RAB sensors and its compass. This is because these are the only sensor-obtained values that our system uses to estimate robot BFVs, and thus relies on for overall performance. We acknowledge that system noise in hardware will not necessarily correspond to noise added in simulation. What we are trying to demonstrate by implementing noise in this manner is that our system is able to retain performance with imperfect information on robot states. The noise applied to robot RAB sensor readings during our experiments (with the exception of those in section Noise Sensitivity) is as follows:

- Linear velocity, simulated Gaussian noise (μ = 0, σ = 5% *d*_*max*_)
- Angular velocity, simulated Gaussian noise (μ = 0, σ = 5% ω_*max*_)

Where *d*_*max*_ is the maximum distance that a robot can travel in a single control step (approximately 0.5*cm*) and ω_*max*_ is the maximum angular distance a robot can travel in a single control-step (approximately 2.4°).

The system parameters used in these experiments are as follows:

- The length of observation period, *o*, during which the assessing robot decides post-diagnosis whether or not the associated recovery action is successful in resolving faulty behavior. *o* = 29.
- The similarity threshold, *s*. When two faults produce an *r*-value using Equation 9 that is greater than *s* they are considered similar for the purpose of diagnosis from memory. *s* = 0.56
- The detection window, *W*. The positive integer *W* represents the number of control-steps over which a faulty robot must be observed to be producing mismatched BFVs before it is declared faulty. This is the accumulation of the data the system has with which to characterize a given fault and attribute to it a signature. *W* = 29
- The proportion of BFVs in *W*, ρ which must be mismatched before a fault is declared. ρ = 0.88

Details of how we arrive at these parameter values can be found in the Supplementary Material for this paper.

Fault memory is written to a circular buffer. In this work we use a buffer with capacity for 18 discrete fault signatures, or three times the number of discrete fault types. 18 places typically ensures that each type of fault has at least one BFV representation in system memory (assuming all fault types have been encountered). Our system is comfortably able to handle memory buffers of this size, and there is no obvious advantage to decreasing the size.

When a fault type and its associated BFVs are stored in the assessing robot's memory, the assessing then passes the information to any other robots it comes within range of, these robots then do likewise and so on. This simulates the swarm of robots sharing and updating fault memory via an *ad-hoc* network. To avoid thrashing, particularly as swarm size increases, robots will record which neighbors they have shared memory with and will not attempt to do so again until at least 1000 control-steps have passed.

## 4. Experiments

The criteria we examine to characterize our system's performance during our experiments (with the exception of those performing collective photo-taxis) are as follows:

- *P*_1_: The total number of faults the system is able to detect
- *P*_2_: The proportion of faults the system is able to diagnose from memory
- *P*_3_: The proportion of attempts to diagnose a fault from memory that fail or are unrecognized by the system
- *P*_4_: The total number of faulty robots the system is unable to resolve and are shut down in an hour of simulated time
- *P*_5_: The average *r*-value between faults, using Equation 9, when diagnosing from memory
- *P*_6_: The average time (in control-steps) it takes the system to detect a fault

For all experiments 100 replicates are performed, from which a median is taken for *P*_1−6_. We selected this number of replicates after performing consistency analysis Read et al. (2012) to find the point at which performing additional replicates does not alter the median value of the entire set. We did this using the SPARTAN package (Alden et al., 2013) on *P*_2_, as we consider this the most indicative measure of overall system performance.

### 4.1. Scalability and flexibility

**Figure 1:**
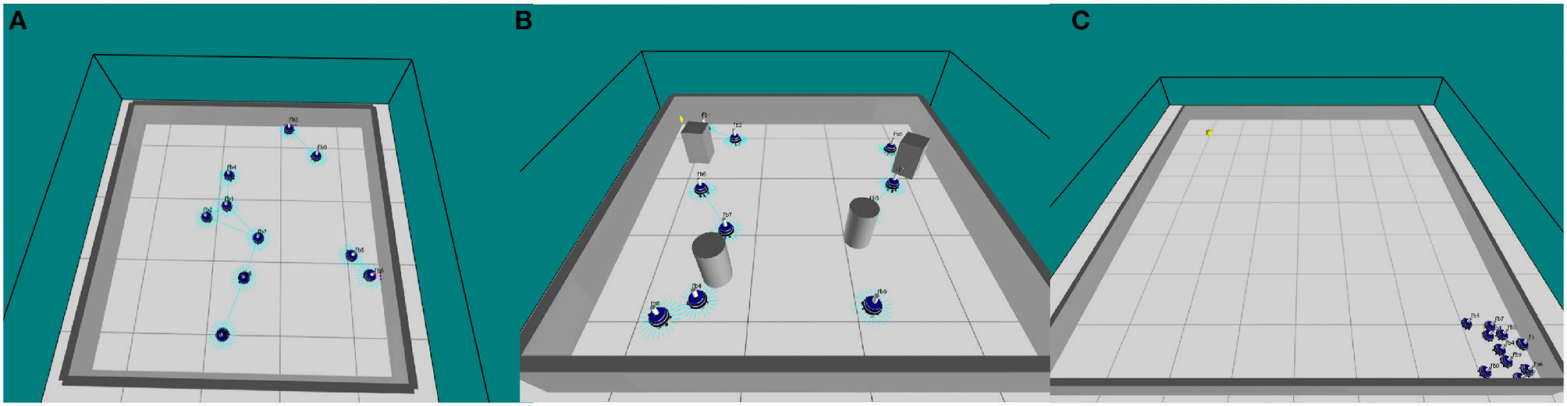
**(A)** ARGoS simulation of 10 marXbot robots performing obstacle avoidance in an empty arena (O'Keeffe et al., 2017a), **(B)** ARGoS simulation of 10 marXbot robots performing obstacle avoidance in a cluttered arena, and **(C)** ARGoS simulation of 10 marXbot robots performing collective photo-taxis in an arena with a beacon in the north-west corner.

To investigate the scalability and flexibility of our system, a swarm of simulated marXbots collectively perform flocking, aggregation or obstacle avoidance behavior in a proportionally scaled enclosed square arena. We set the ratio of robots to square meters at 1:1.6 (see Figure 1A).

These experiments run for 1 h of simulated time (36,000 control-steps). The swarm's normal behavior is updated randomly between flocking, aggregation or dispersion at 5,000 control step intervals. Each robot has a 0.1% probability of having a random fault injected at each control-step. A maximum of 50% of the robots in a swarm can be faulty at one time, so that there were never more faulty robots than the swarm could provide assessors for. Our approach here means that every time a test is run, the swarm exhibits a variety of behaviors and is subjected to a variety of faults—usually exhausting all possible combinations. For this reason it is proposed that the results obtained satisfy the conditions for swarm flexibility (Şahin, 2004).

#### 4.1.1. Results and discussion

The performance of our diagnosis system on robot swarms of different sizes is displayed in Table 1. The total number of faults detected, *P*_1_, is removed from the table as this will increase proportionally as swarm size increases, and thus is not an indicator of the system's scalability.

**Table 1:** Performance of system for swarms of varying sizes.

**Swarm size**	***P*_2_**	***P*_3_**	***P*_4_**	***P*_5_**	***P*_6_ (control-steps)**
10	0.68	0.04	0	0.87	444
40	0.76	0.03	0	0.91	572
90	0.76	0.04	0	0.90	474
160	0.74	0.05	1	0.90	592

*Results are taken from the median values of 100 experimental replicates*.

Table 2 shows how the performance of our swarm varies when obstacles are added to the arena, and when fault memory gathered from the swarm in an empty arena is imported into a swarm in an arena with obstacles (see Figure 1B). The purpose of the latter is to demonstrate that our proposed system is flexible to varying or dynamic environments.

**Table 2:** Performance of system for robot swarms in different environments.

**Arena type**	***P*_1_**	***P*_2_**	***P*_3_**	***P*_4_**	***P*_5_**	***P*_6_ (control-steps)**
No obstacles	30	0.68	0.04	0	0.87	444
Obstacles	30	0.68	0.04	0	0.87	403
Obstacles (imported memory)	30	0.93	0.03	0	0.91	413

*Results are taken from the median values of 100 experimental replicates*.

Table 1 shows the proportion of faults successfully diagnosed from memory, *P*_2_, increases slightly between swarm size 10 and 40. *P*_2_ then stabilizes between swarm sizes of 40 and 90, in which no significant trend is observed. Performing a Wilcoxon rank-sum test Haynes (2013) on the data distributions of *P*_2_ for swarm sizes of 40 and 90 produced a value *p* = 0.98, where *p*>0.05 indicates that two data distributions are statistically similar. The small drop in *P*_2_ for swarm sizes of 160 is discussed later in this section. The reason for the increase in *P*_2_ between swarm sizes of 10 and 40 is because of the tendency to diagnose from memory more frequently as each experiment run goes on. In each experiment the system reaches a point of familiarity with the faults it can be subjected to, after which it can diagnose subsequent faults from memory in a majority of cases. We previously demonstrated in O'Keeffe et al. (2017b) that the majority of diagnoses occur in the latter two thirds of the hour-long experiments for a swarm with 10 robots—this is approximately where the system reaches its point of familiarity. The rate at which the system is able reach this point will be affected by how often the system encounters faults and how quickly it can resolve them. Reaching the point of familiarity will be accelerated as swarm size increases (as this will inherently increase the frequency of faults occurring) and, combined with the increased overall amount of faults encountered by the system, contributes to the increase in *P*_2_ observed between swarm sizes 10 and 40. This would suggest that the saturation point of the system reaching the point of familiarity with respect to increasing swarm size lies between 10 and 40.

Table 1 does not display a clear trend between swarm size and the proportion of unsuccessful attempts to diagnose faults from memory, *P*_3_. The distribution of *P*_3_ does not change significantly for swarm sizes of 10 and 90, performing a Wilcoxon rank-sum test on these distributions produces a value *p* = 0.152. *P*_3_ is at its highest for a swarm size of 160. We would attribute this, coupled with the slight drop in the proportion of faults successfully diagnosed from memory, *P*_2_, to the increased probability that a faulty robot will find itself near the center of a robot cluster at the time it is being observed by an assessing robot. If the robots neighboring the faulty robot are unable to disperse or otherwise move out of the faulty robot's path it may disrupt the assessment process.

The total number of faults that are unresolvable, *P*_4_, is 0 between swarm sizes of 10 and 90. *P*_4_ increases from 0 to 1 for swarm size 160, which we would attribute to the vastly increased swarm size increasing the probability of the circumstances in which a faulty robot is unresolvable occurring within 1 h. The average *r*-value between faults, *P*_5_, and the average time taken to detect faults, *P*_6_, do not display a clear trend with increasing swarm size.

Table 1 reveals slight fluctuation in performance with varying swarm size. These fluctuations are observed over an order of magnitude, whilst *P*_2_, the most indicative measure of performance, remains within approximately 90% of its observed maximum. Based on this, we would argue our system to be stable—and thus scalable—for practical swarms of between 10 and 160 robots.

Table 2 shows that the only difference in median average performance between a swarm in an empty arena and a swarm in a cluttered arena is a reduction in the average time taken to detect faults, *P*_6_, of approximately 9%. For swarms in an arena with and without obstacles, there is no significant difference between the distributions of performance criteria *P*_1−4_ when subjected to a Wilcoxon rank-sum test (*p*-values 0.33, 0.33, 0.31, 0.81, 0.55, respectively). When fault memory from the swarm in an empty arena is imported to a swarm in an arena with obstacles, there is a large improvement in the proportion of faults successfully diagnosed from memory, (*P*_2_), approximately +37%, and the proportion of attempts to diagnose faults from memory that were unsuccessful, *P*_3_, approximately −25%. This is because the system begins the experiment already at the point of familiarity with the faults that will be injected and so is able to diagnose a greater proportion from memory—even though the system's gathered memory originates from the empty arena. This suggests that there is little difference between the fault data gathered from robots performing in empty arenas and those in cluttered arenas. The data in Table 2 suggests that our system is flexible and robust to environments with varying degrees of complexity, so long as they do not inhibit the swarm from performing normally.

### 4.2. Noise sensitivity

These experiments again use an identical setup to that described in the flexibility and scalability experiments, with the exception that noise applied to the system is varied.

We use Latin Hypercube Analysis to test our systems performance for varying levels of noise on the linear and angular velocity readings obtained by robot RAB sensors. This is a statistical technique for sampling multi-dimensional data distributions, two dimensional in this instance. We use the SPARTAN R package Alden et al. (2013) to perform the sampling for Latin Hypercube Analysis, which allows us to randomly generate 500 parameter sets, where each parameter value is unique in the set, in the following ranges:

- RAB sensor linear velocity reading, simulated Gaussian noise (μ = 0, 0 < σ < *d*_*max*_)
- RAB sensor angular velocity reading, simulated Gaussian noise (μ = 0, 0 < σ < ω_*max*_)

Again, we perform 100 replicates for each parameter set and take the median value for performance criteria *P*_1−6_.

#### 4.2.1. Results and discussion

**Figure 2:**
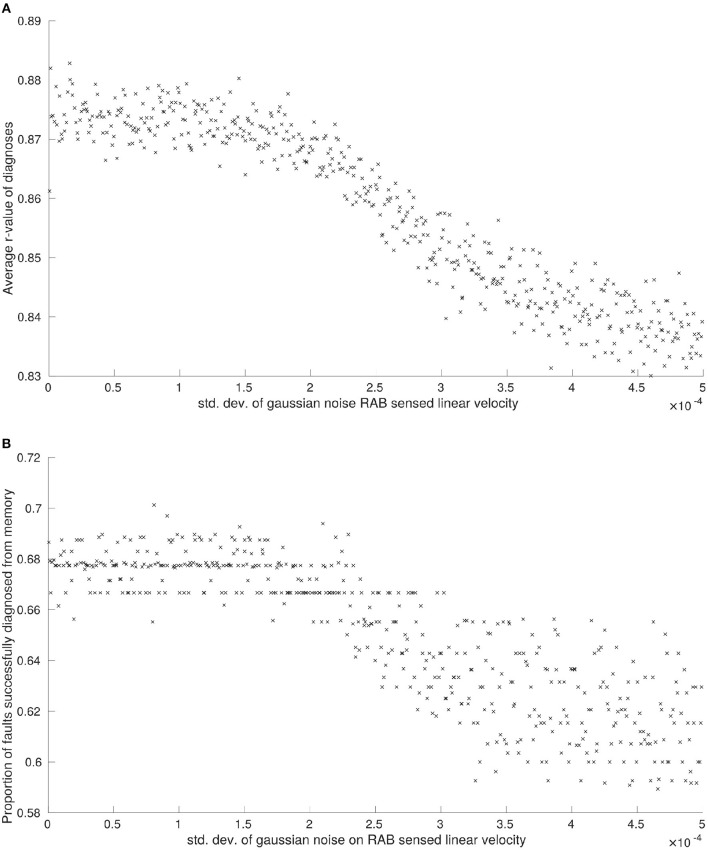
**(A)** The effects of noise on RAB sensed linear velocity on median r-values between diagnoses, and **(B)** the effects of noise on RAB sensed linear velocity measurements on the proportion of faults successfully diagnosed from memory.

**Figure 3:**
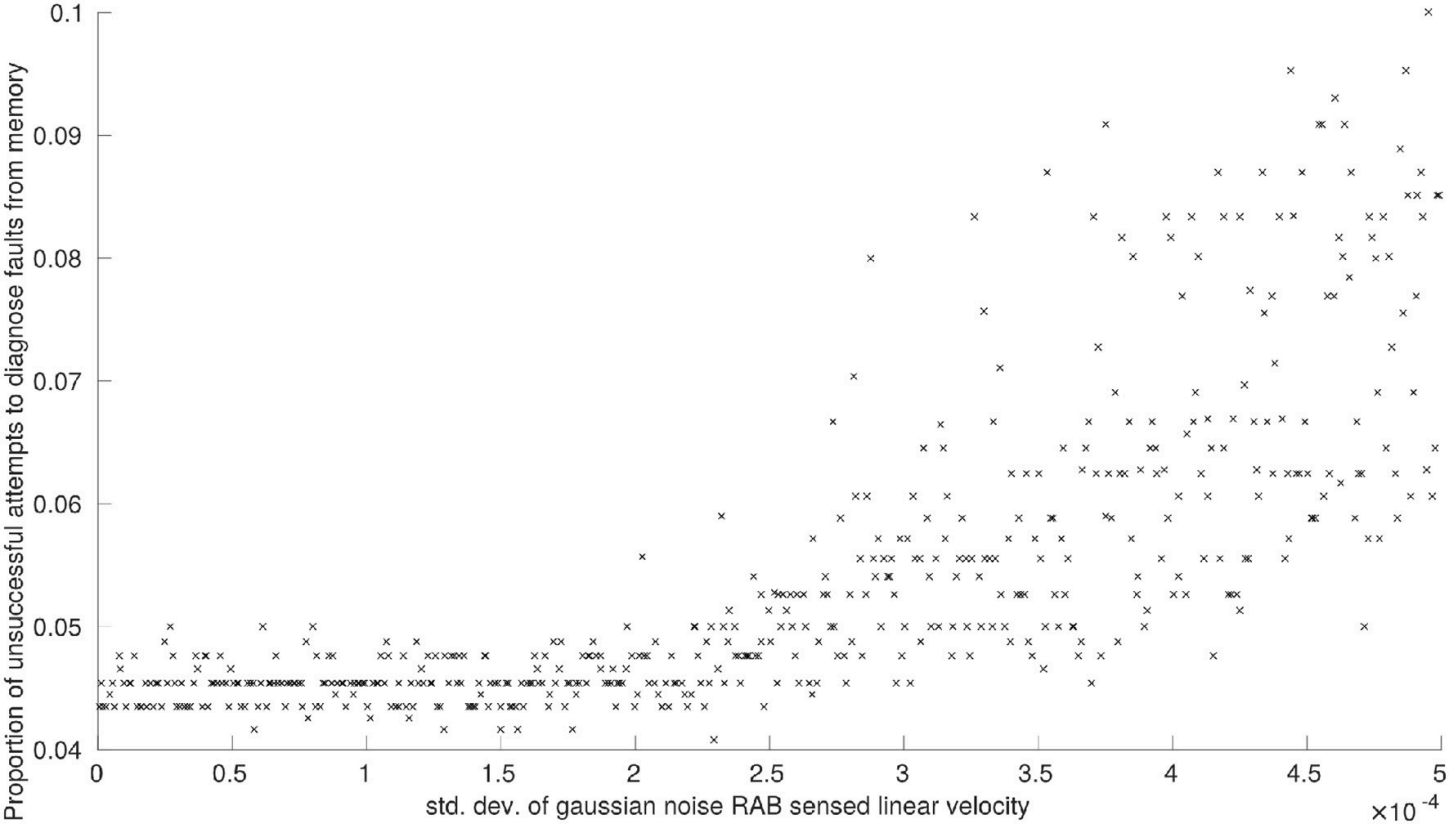
The effects of noise on RAB sensed linear velocity measurements on the proportion of unsuccessful attempts to diagnose faults from memory.

Again, we only plot the results of these experiments where there is a correlation to be observed between the standard deviation of applied Gaussian noise and the performance criteria (*P*_1−6_). This is displayed in Figures 2, 3.

We did not observe a visible correlation between the standard deviation of Gaussian noise applied to RAB sensor readings and the average time taken to detect faults, *P*_6_, as we would expect.

The average *r*-value between faults, *P*_5_, was noticeably affected by noise applied to linear velocity estimations obtained from RAB sensors (see Figure 2A), although this is observed over a comparatively small scale. Interestingly, there was no observable correlation between *P*_5_ and the noise applied to RAB estimated angular velocity. The reason for this observed robustness is that angular velocity is only able to affect a single robot feature $F_{5}^{e}$. The maximum effect that noise can have on a feature is setting it to an incorrect state. Because our features are set by thresholds, in order for the true state to be altered by system noise, the noise needs to be of a significant magnitude *and* of the appropriate sign. As the simulated noise we generate on our sensors is normally distributed, this means that the probability that a feature state is correct will always be greater than that of it being incorrect, no matter how large we set the standard deviation of noise to be. These factors combined mean that the overall change in robot BFVs is comparatively small.

We observe the same trend for proportion of faults successfully diagnosed from memory, *P*_2_, and proportion of attempts to diagnose faults from memory that are unsuccessful, *P*_3_. Noise applied to RAB sensed linear velocity has a clear correlation with system performance (see Figures 2B, 3, respectively) whilst noise applied to RAB sensed angular velocity was not observed to have any noticeable effect by comparison.

The total number of unresolvable faults, *P*_4_, was unaffected by noise applied to sensor readings. This is primarily because a robot is only declared faulty when it is consistently unable to pass diagnostic tests *T*_1_−*T*_6_. Of these diagnostic tests, the only tests reliant on feature observations that could be disrupted by sensor noise are *T*_4_ and *T*_5_ (constituting a third of all faults encountered by the system on average) and, even with maximum sensor noise applied, we have already discussed how there is a greater probability that features will remain in the correct state at any given point in time. Although increasing sensor noise will increase the probability that diagnostic tests need to be run (Figures 2B, 3), there is still only a slim increase in overall probability that a faulty robot will be deemed unresolvable.

We have not examined how sensor noise would impact fault detection in this work. Based on the effects of noise on RAB sensor readings, (Figure 3), we can predict that increasing sensor noise (at least on the virtual range sensors) would drastically increase the amount of false-positives encountered by the system in a scenario where fault detection was more realistically implemented. Although our results demonstrate that the system would generally be capable of resolving these scenarios, the amount of time spent with one or more robots performing diagnostic routines would increase in sympathy—which may cause problems in time-critical scenarios or where a certain number of functioning robots are required.

### 4.3. Collective photo-taxis

In these experiments we again consider a swarm of simulated marXbots, this time performing collective photo-taxis behavior. Our implementation of this behavior is similar to that used by Bjerknes and Winfield (2013) whereby robots in the swarm will attempt to aggregate whilst avoiding collisions with one another.Robots who are closer to a beacon will avoid neighboring robots at greater distances than those who are furthest away (robots usually turn to avoid collision at distances < 0.3*m*, for these experiments the robots closest to the beacon will turn away from neighbors at distances < 0.35*m*). The emergent effect of this behavior is that the swarm gravitates toward the beacon.

Here we compare the performance of a normally functioning swarm, a faulty swarm without active fault tolerance and a faulty swarm with active fault tolerance. Our experiments use a swarm of 10 marXbots in an enclosed arena (6*m* x 6*m*), where robots begin in the south-east corner of the arena and are drawn to a light source at the north-west corner (see Figure 1C).

Our diagnosis system is implemented in the same manner as previously. Robots that are engaged in the diagnostic process are unable to participate in collective photo-taxis, however the assessing robot will still repel nearby neighbors, preventing the diagnostic process from anchoring the swarm. Faults are injected into between 1 and 5 robots after the first 500 control-steps of each experiment, as this was found to be the point at which swarm behavior had typically stabilized (in flocking or aggregation behavior, for example, one or more robot clusters will have formed after 500 control-steps have passed).

The performance criteria we examine in these experiments are the times taken for the first member of the swarm to reach the beacon, the time taken for half the members of swarm to reach the beacon, and the time taken for all members of the swarm to reach the beacon, and the average distance of all members of the swarm at these times, respectively. An individual robot is considered to have reached the beacon when the distance between the two is < 0.8*m*.

We use the performance of a normally functioning swarm as a point of reference, which is displayed in Table 3.

**Table 3:** Normally functioning swarm performance of collective photo-taxis.

	**Time (control-steps)**	**Avg. dist. from beacon (meters)**
1st Robot	24,388	1.4275
Half Robots	25,548	0.8517
All Robots	26,909	0.4746

*Results are taken from the median values of 100 experimental replicates*.

We examine the performance of a swarm where 1−5 robots are faulty as a proportion of the performance of a normally functioning swarm, where we obtain our value for proportional performance using Equation (10).

(10) $$\begin{array}{l} \text{Proportional Performance}=\frac{1}{3}\overset{3}{\underset{i=1}{\sum }}\frac{dt_{i_{0}}-dt_{i}}{dt_{i_{0}}} \end{array}$$

Where *dt*_1−3_0__ and *dt*_1−3_ are the average distances between the normally functioning swarm and the beacon and the average distances between a faulty swarm and the beacon after 24388, 25548 and 26909 control-steps, respectively (these values are taken from Table 3).

We compare the proportional performance of robot swarms with and without our proposed diagnosis system.

#### 4.3.1. Results and discussion

**Figure 4:**
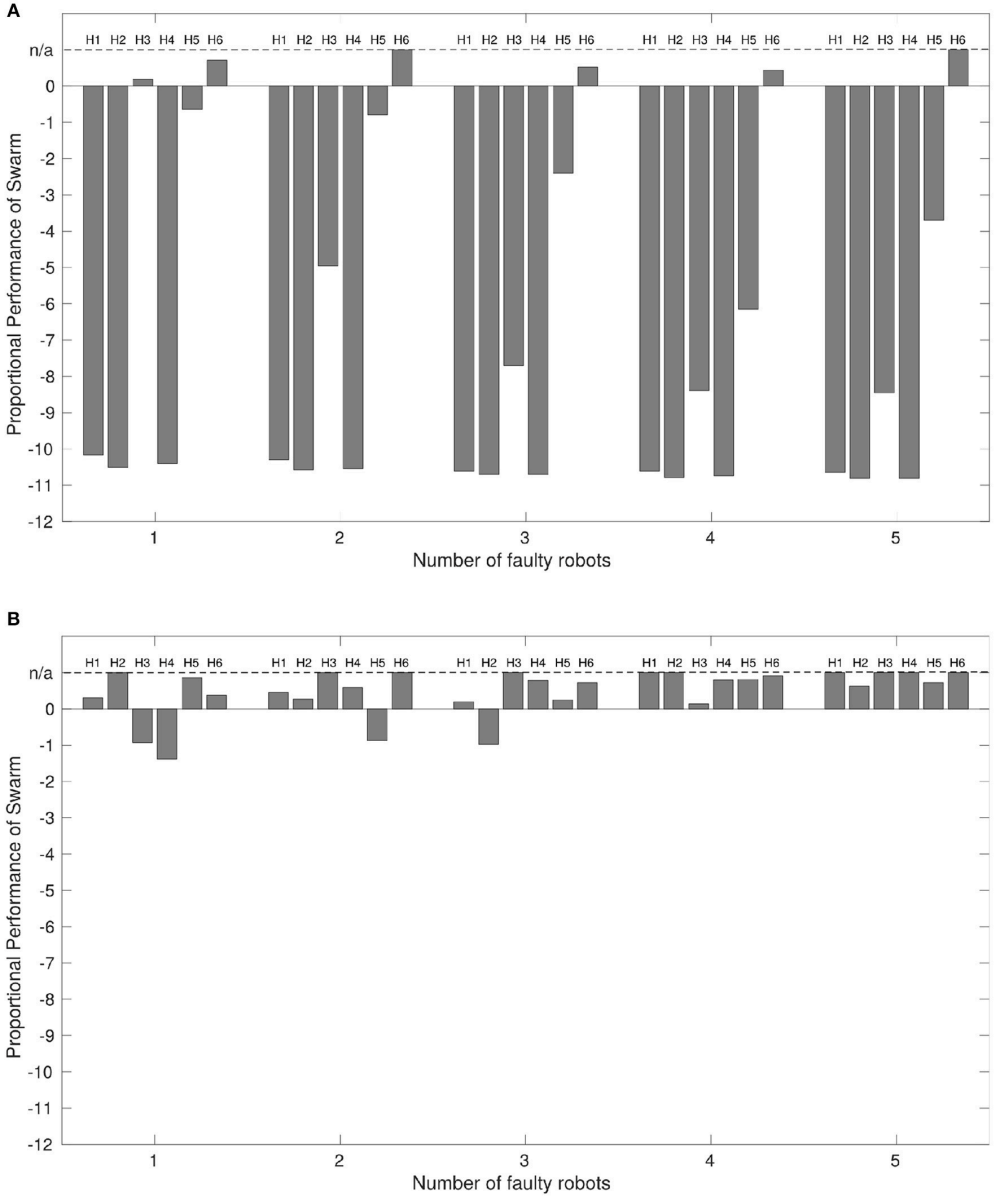
**(A)** The performance of a robot swarm with no active fault tolerance exhibiting the collective photo-taxis when a varying proportion of robots are subjected to a variety of faults, and **(B)** The results of the same experiment with active fault tolerance. *x* = 0 indicates a performance equivalent with a normally functioning swarm. Results are taken from the median values of 100 experimental replicates. The dashed line at *x* = n/a indicates that proportional performance is not applicable because the entire swarm reached the beacon in < 24,388 time-steps, meaning that median average distances at *t* ≥24, 388 control-steps could not be taken.

Figure 4A displays the proportional performance of the robot swarm performing the ω-algorithm with up to 5 faulty robots where we do not implement our diagnosis system. Figure 4B displays the proportional performance of the same swarm in the same conditions where we have implemented our diagnosis system.

Where the output of Equation (10) is < 0 indicates the faulty swarm has not performed as well as the normal swarm. Where the output is equal to 0 indicates an equivalence in performance between faulty and normal swarms. Where the output is >0 indicates that the faulty swarm has performed better than the normal swarm. In some cases, the faulty swarm outperforms the normal swarm by such a large margin that comparing the distances at the times of interest listed in Table 3 loses applicability. In this case the value of some or all parts of *dt*_1−3_ go to 0, returning a proportional performance value of 1. As proportional performance should only ever approach 1 asymptotically, performance in these instances is labeled as *n*/*a* in Figure 4 for clarity.

With the exception of some cases of partial sensor failure, *H*_6_, the performance of the swarm with any number of faulty robots is worse than for a normally functioning swarm, which corresponds with the findings of Winfield and Nembrini (2006).

Interestingly, Figure 4A shows that the presence of some faults, most notably partial sensor failure, actually improves overall performance from that of a normally functioning system. The reason for this is that robots afflicted with partial sensor failure will not be inclined to aggregate toward the center of a swarm if the center of mass is behind them. This means that the faulty robots will press ahead, whilst normally functioning robots are consequently able to move toward the beacon at a faster rate.

We can now compare the performance of the swarm under the same conditions when we apply our diagnostic system. The results are shown in Figure 4B.

Figure 4B shows that, again with exception of some cases of partial sensor failure, *P*_6_, the presence of our diagnosis mechanism results in a large improvement in overall system performance where faulty robots are present. Additionally, in a number of cases overall performance is again better than that of a normally functioning swarm. This is because when two robots are involved in a diagnostic routine, they will repel other normally functioning robots in the swarm, allowing them to continue toward the beacon unhindered and at a faster rate brought about by a smaller local swarm size (this is only true for local swarm sizes with 3 or more robots, however this is most likely the case in these experiments given that all robots start experiments within local sensing range of each other Figure 1C).

We propose that Figure 4 validates our system in the context of the original proposal for active fault tolerance in swarms by Winfield and Nembrini (2006). Our results demonstrate a clear improvement in swarm performance in most cases where active fault tolerance mechanisms are present.

## 5. Limitations

We acknowledge that our system is heavily dependent on reliable communication between robots. For our system, robots will need to communicate their own BFV states with each other, as well as sharing their memory of previous fault characterizations. If this data is lost, delayed, sent or received in the wrong order or otherwise not as it should be, this will affect system performance. When robots communicate their BFV states it must be done in real time with as little delay as possible. If this is hindered, we can predict two possible outcomes; a robot will be unable to make a comparison of proprioceptively and exteroceptively estimated BFVs at that point in time, in which case any active diagnostic processes will have to pause until up-to-date BFV information becomes available again; or, robots will have to use the most recent BFV data irrespective of whether or not it accurately describes a robot's present state—which is tantamount to system noise in this case, the effects of which we have already shown to be detrimental to overall performance. Similarly, if memory data is lost or corrupted, the system will either have non-representative fault characterizations—which will lead to an increase in failed diagnosis attempts—or it will simply have no representation at all in these scenarios, in which case it will require the time-consuming diagnostic tests to be run more frequently.

We also acknowledge that the work presented in this paper only considers individual robot faults that can be considered orthogonal to each other. In real world scenarios it is probable that multiple faults may occur at once or in sequence, or that the effects of one type of fault may be indistinguishable from the effects of a different type of fault by their BFV signatures—we observed this in our earlier work (O'Keeffe et al., 2017a), however it might not always be possible to get around these problems with the addition of new features. Additional problems may occur if faults do not explicitly manifest in robot software/hardware, but in the interaction space between robots. Although it is beyond the scope of the work, as AIS in robots develop, it is probable that Trusted Autonomy (Abbass et al., 2016) will become an increasingly prevalent consideration for fault tolerant systems. The system described in this work is not equipped to examine or address this problem space, and to modify it do so would require an additional dimension of FDDR to be integrated.

In the wider context of achieving fault tolerant swarms, another limitation of our system, and possibly of the process of fault diagnosis in general, is the dependence on reliable fault detection. This is indicated by the results of our noise sensitivity analysis, and in the parameter sensitivity analysis in the Supplementary Material. Without a reliable means of autonomous fault detection, our system is impaired in its ability to demonstrate learning and memory functionality as we would like. Furthermore, fault recovery at a finer scale than the replacement of an entire robot is a problem that is beyond the capabilities of any swarm robotic platforms known to the authors at time of writing. Until the functionality of swarm robotic hardware increases such that basic recovery actions—such as those described in this work or similar—are possible, the path to achieving fault tolerant swarms will be confined to the realm of concept.

## 6. Conclusions and future work

In this work we have presented a novel approach toward autonomous fault diagnosis in robot swarms. We demonstrated our system with specifically chosen parameter values to be flexible and scalable, being able to diagnose faults from memory in 68–76% of cases on average, depending on the size of the robot swarm. Our system also displayed a low rate of misclassification (3–5% on average) and a general tolerance to imperfect robot state information.

We were able to demonstrate a clear improvement in performance when our diagnosis system was implemented on a swarm performing collective photo-taxis and subjected to partial faults. This improvement supports the case for active fault tolerance proposed by Bjerknes and Winfield (2013) and also represents a viable solution for artificial immunity in robot swarms, as our method for autonomous fault diagnosis should be compatible with existing methods for autonomous fault detection and, eventually, autonomous fault recovery.

We acknowledge that truly optimal system parameters, and the extent of some system limitations—such as dependence on reliable communication—cannot be known until fully integrated FDDR is implemented in a hardware robot swarm. Our future work will be toward addressing this problem by implementing our diagnosis system as faithfully as possible on a swarm robotic platform in hardware.

## Data availability statement

The datasets produced and analyzed for this study can be found in here: https://zenodo.org/record/1467221#.W8n7mvn27mE.

## Author contributions

JO: performed all experiments and obtained all results described; Principal author of this paper; DT: critical revisions; AM: critical revisions; JT: critical revisions.

### Conflict of interest statement

The authors declare that the research was conducted in the absence of any commercial or financial relationships that could be construed as a potential conflict of interest.
