# Supplementary material for: Adaptive Online Fault Diagnosis in Autonomous Robot Swarms
Source: Front Robot AI. 2018 Nov 30;5:131. doi: 10.3389/frobt.2018.00131 (PMC7805982; doi:10.3389/frobt.2018.00131)
Supplement: Supplementary file 1 [file Data_Sheet_1.pdf]

Adaptive Online Fault Diagnosis in Autonomous Robot Swarms (Supplementary Data)

James O'Keeffe [1,*], Danesh Tarapore [2], Alan G. Millard [1] and Jon Timmis [1]

[1] *University of York, Department of Electronic Engineering, York, UK*
[2] *University of Southampton, School of Electronics and Computer Science, Southampton, UK*

Correspondence*:
James O'Keeffe
jhok500@york.ac.uk

ABSTRACT

This document is intended as supplementary data to the paper 'Adaptive Online Fault Diagnosis in Autonomous Robot Swarms'. We describe here the process sensitivity analysis that informed our parameter selection for the experiments in the main paper.

1 SUPPLEMENTAL DATA

1.1 Parameter Sensitivity Analysis

Using an identical setup to that detailed in our scalability and flexibility experiments in section 4.1, we observe the sensitivity of overall system performance to variable system parameters.

The purpose of these experiments is twofold. Firstly, we want to identify a baseline parameter configuration for our own future work. Secondly, we aim to shed a general light on where certain parameter configurations become unacceptable, as parameters such as the period of observation, the detection window or variants of these will likely be common to future approaches to fault diagnosis in swarms.

We used the SPARTAN to generate 500 parameter sets for the four parameters in the following ranges:

- Observation period, $1 < o < 100$ (control-steps)
- Similarity threshold, $0 < s < 100$.
- Detection window, $1 < W < 500$. (control-steps)
- The proportion of mismatched BFVs in W, $0.01 < \rho < 1$.

Once again, 100 replicates are performed for each parameter combination, from which a median is taken for performance criteria P_{1-6}.

1.1.1 Results & Discussion

Results from these experiments are only plotted in this work where there is a correlation to be observed between a parameter value and performance criteria (P_{1-6}).

Sensitivity to observation period

The value of the observation period, o, predictably, did not effect the average time taken for the system to detect faults, P_6. More surprisingly, the value of o did not effect the total number of faults detected by the system, P_1. We believe this to be because of two factors; the decentralised nature of our system means that faults can be recovered in parallel i.e. the system does not need

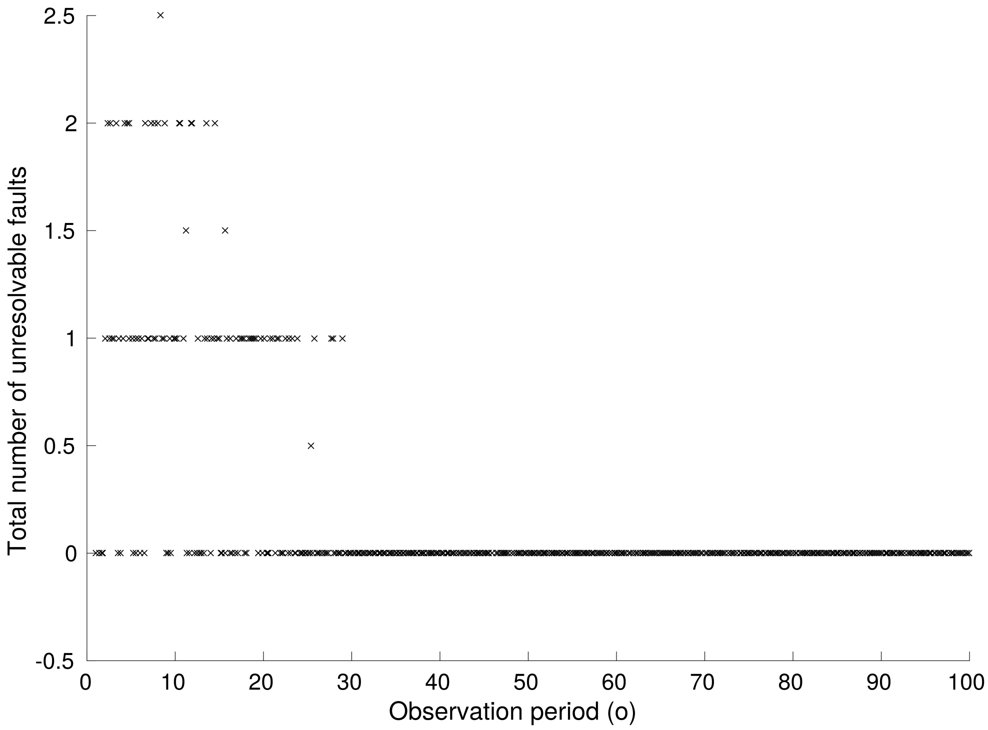

Figure 1. The median total number of unresolvable faults, P_4, over one hour of simulated time against the value of the observation period, o. Non-integer values indicate a median value between two integer values, rather than 0.5 unresolvable robots.

to wait until one fault is resolved before it can begin working on another, and secondly; the 100 control-step maximum value of o is a short amount of time compared to the 36000 control-step experiment length.

The value of o most prominently affected the total number of faulty robots that the system is unable to resolve, P_4 (see Figure 1). In the experiments performed, diagnostic tests failing to resolve the fault occurred under one scenario; where a faulty robot became stuck in a corner or against a wall or object. In these circumstances the fault could have been diagnosed correctly, either from memory or the diagnostic tests, and the fault resolved. However, the subsequent observations of the robot would lead the assessing to believe the fault had persisted. This is because the faulty robot would try to move normally but be obstructed by the walls of the arena. How long the robot is observed for directly corresponds to the likelihood of this scenario occurring; the larger the value of o, the more opportunity a stuck robot has to become unstuck and demonstrate its normal function.

The the value of o also has a somewhat less pronounced effect on the proportion of faults successfully diagnosed from memory, P_2, the proportion of unsuccessful attempts to diagnose faults from memory, P_3, and the average r-value produced between faults P_5. For all three properties, o values of less than 5 tend to severely reduce performance. Above this value, however, there is no observable correlation as o increases. To minimise the time spent in the diagnostic process and the number of robots lost and to maximise reliability of diagnoses from memory, we therefore set $o = 29$.

Sensitivity to similarity threshold

In our experiments we observed the similarity threshold, s, to have no effect on the total number of faults detected, P_1, or the average time taken to detect them, P_6, as we would predict. The value of s was not observed to have any obvious correlation with the total number of unresolvable

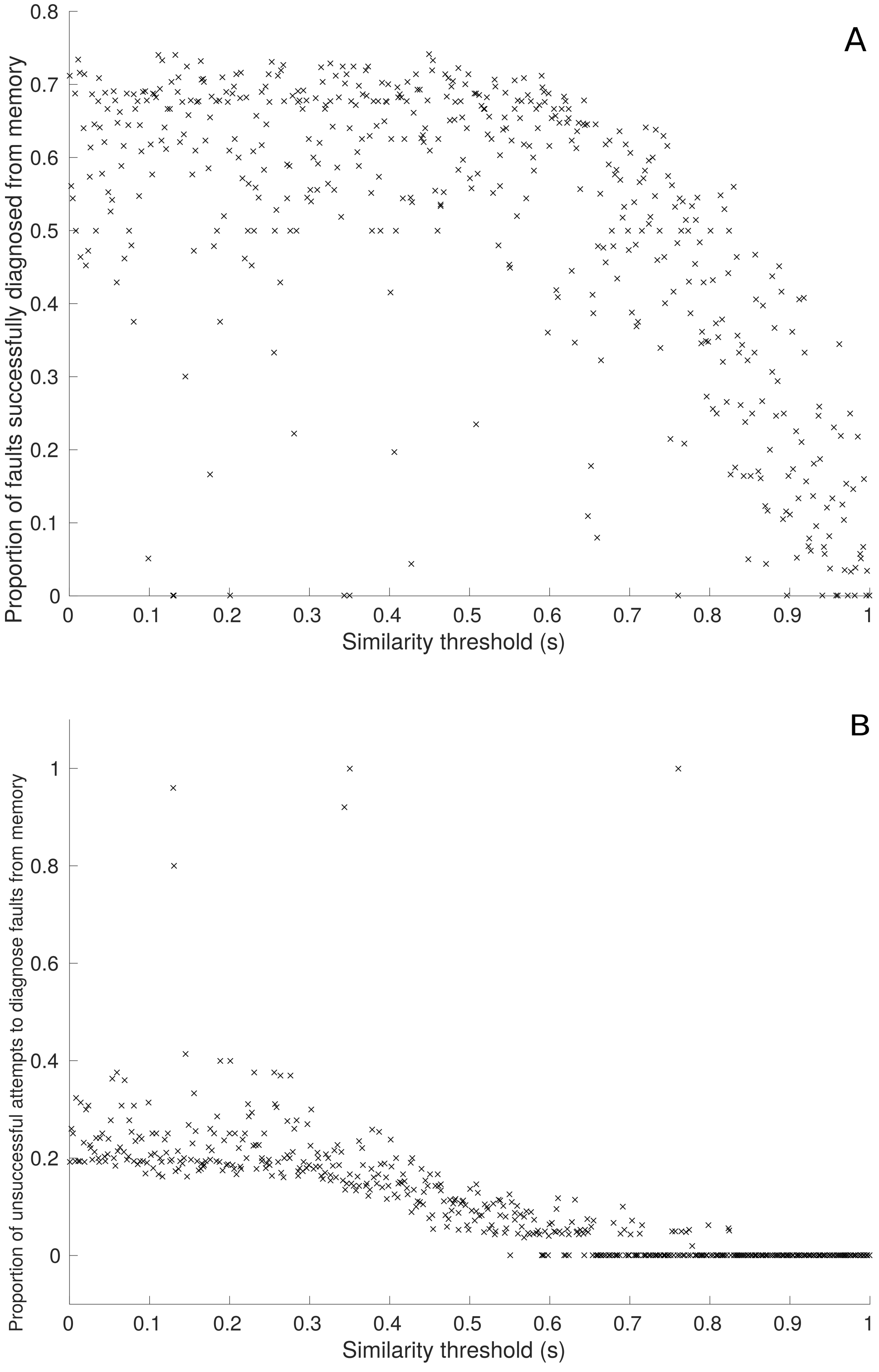

Figure 2. **A**: The median proportion of faulty robots the system was successfully able to resolve from memory against the value of the similarity threshold, *s*, and **B**: the median proportion of attempts by the system to diagnose from memory that failed or were not recognised as successful against the value of the simulation threshold, *s*.

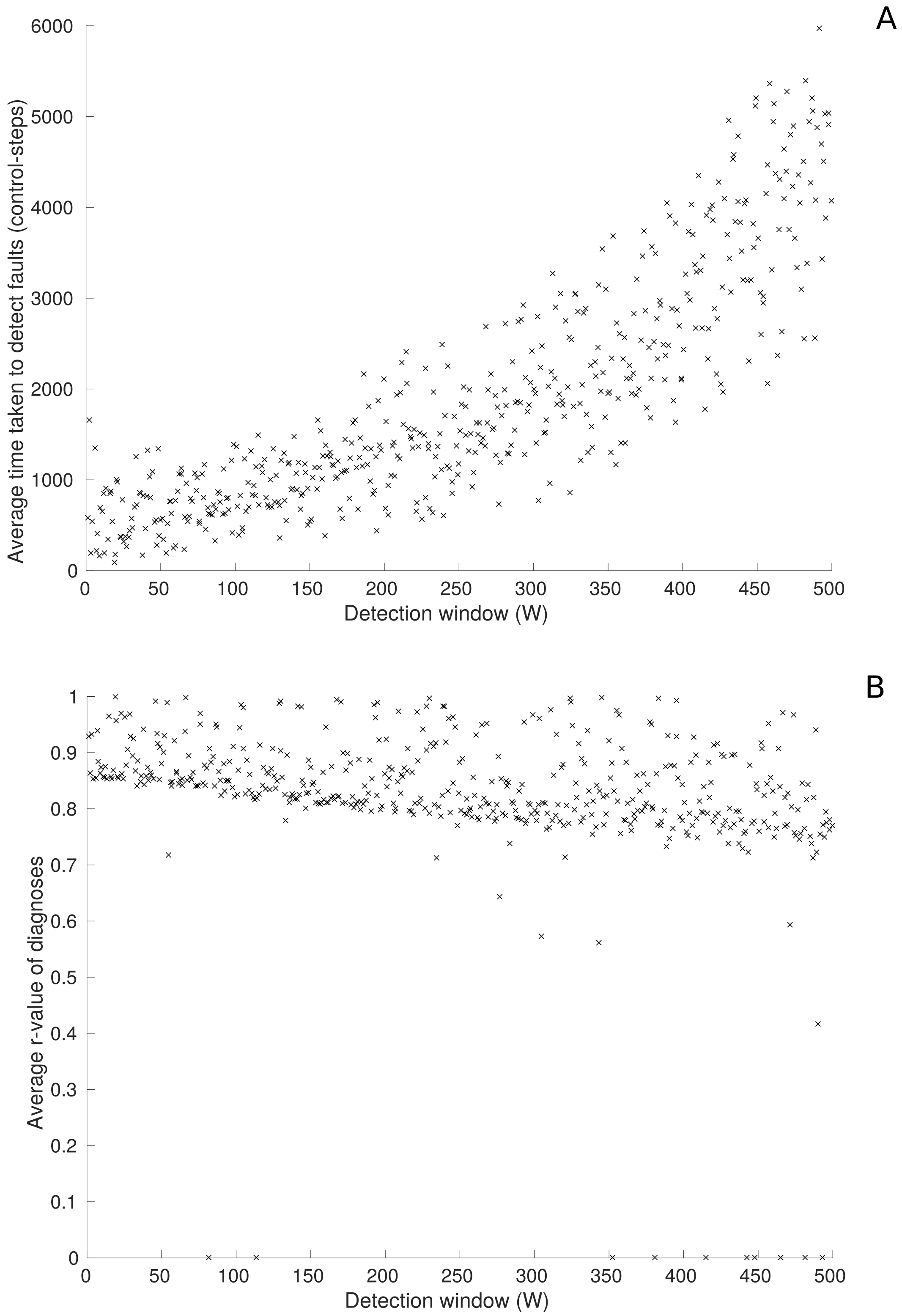

Figure 3. **A**: The median time taken to detect faults against the value of the detection window, W, and **B**: the median r-value of successful diagnoses against the value of the detection window, W.

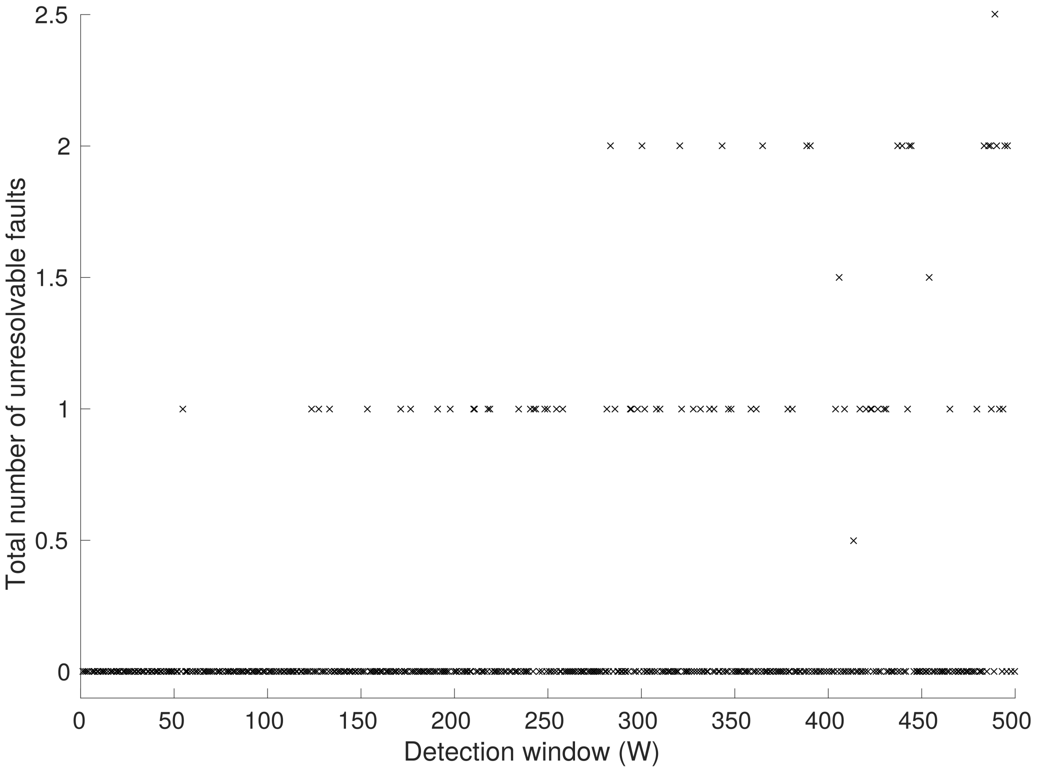

Figure 4. The median total number of faulty robots the system was unable to resolve over one hour of simulated time against the value of the detection window, W. Non-integer values indicate a median value between two integer values, rather than 0.5 unresolvable robots.

faults, P_4, as this is caused by the circumstances in which the diagnostic tests are run, rather than how well two faults correlate. As s increases, the average r-value between faults, P_5, also increases. However, this is simply because lower r-values are removed from the values which are averaged.

As s approaches 1, the proportion of faults successfully diagnosed from memory, P_2, remains largely unresponsive until $s > 0.55$, at which point it rapidly declines (suggesting that the average r-value for all fault, behaviour and parameter combinations is approximately at this point) Figure 2A. Therefore to maximise the number of faults the system can diagnose from memory, and minimise the time spent in the diagnostic process, the value s should be minimised. However, as s approaches 1, the proportion of unsuccessful attempts to diagnose faults from memory, P_3, decreases in sympathy, averaging 0 for all other parameter combinations at $s > 0.825$ Figure 2B. The value s will therefore be greater than 0.825 in order to minimise time wasted on performing incorrect recovery actions.

The goal here is to find the value of s that will provide optimal cost efficiency between running more diagnostic tests and performing wasteful recovery actions. For a real world scenario, this will depend on a number of circumstances such as the time-sensitivity of a particular task, and the number of replacement parts available to the swarm. If we assume that there is an ample stock of replacement robot parts, this becomes a time-optimisation problem, specifically between the time taken to perform recovery actions and the time taken to perform the diagnostic tests. We examined the time taken to perform recovery tests in our previous work, O'Keeffe et al. (2017), however, as we stated then, the time taken to perform recovery actions will vary for different swarm robot platforms, and cannot be known in the context of our work until the specific recovery actions we described in section 3 have been investigated for an appropriate robot platform. We would argue that the best we can do at this stage is minimise the number of attempts to diagnose

a fault from memory that fail to resolve the fault. Figure 2B shows that for $s >= 0.56$ there are parameter combinations that result in an average of $P_3 = 0$, suggesting that for combinations with $s >= 0.56$ where there are failed attempts, it is because of an alternative parameter value. We therefore set the value of s to 0.56. Conveniently, this allows us to theoretically retain a value of P_2 that is close to the maximum potential value.

Sensitivity to detection window size

Our experiments showed that as the detection window size, W, increases, so too does the time taken to detect faults, P_6, (see Figure 3A), which consequently decreases the total number of faults detected by the system, P_1. In order to maximise how quickly our system is able to resolve the faults we should minimise the time spent observing the fault prior to investigating.

We observed that as the detection window size, W, increases, so too does the proportion of unsuccessful attempts to diagnose faults from memory, P_3, and that the proportion of faults successfully diagnosed from memory ,P_2, decreases. This trend arises from the tendency of a larger detection window to reduce the average r-value of diagnoses, P_5 (see Figure 3B).

The reduced average r-value of diagnoses, P_5, with increasing values of W is caused by greater opportunity for what could be considered outliers in robot behaviours to be observed in the fault's characterisation. To illustrate what we mean by this; consider two instances of a robot with a partial motor failure. The robot is detected when it is observed to be moving in an arc when attempting to move in a straight line. If the faulty robot is observed for a very short period of time, it is likely that this effect will be observed for the entire duration each time, resulting in a very high r-value between the two faults. If the effect is not observed, the faults will not be detected. If it is only observed in one instance, the two faults will then have such low r-values that they will not be recognised as the same fault and therefore not eligible for diagnosis from memory. If the faulty robots are observed for a long period of time, it is far more likely that the arcing effect will be observed for a majority of the time, but not the entirety – resulting in the lower r-values we observe in Figure 3B.

There is also a positive correlation between W and the total number of unresolvable faults, P_4, (see Figure 4). Because this effect arises from the environmental circumstances of the faulty robot, we attribute this correlation to the increased time a robot will spend being faulty before being detected as such – which increases the probability that these circumstances arise.

All analysis of W suggests that its should be minimised. The value of W could theoretically be set to 1 in a noiseless system, and our results reveal no obvious drawback to using the smallest possible value of W. However, we have not thoroughly examined how the value of W will affect fault detection, particularly false-positive fault detection. Using the method of fault detection described in this paper, a low W value combined with any system noise or delay whatsoever would certainly result in large numbers of false-positive detections – resulting in time and resources being wasted by the system on performing diagnostic tests and wasteful recovery actions. Although it is impossible to know precisely how the value of W will effect system efficiency until fault detection and diagnosis are properly integrated, we can say that the optimal value will strike a balance between maximising the r-values between faults and minimising time taken to detect faults and the frequency of false-positives. In our analysis of the observation period, o, we ascertained 29 control-steps to be the minimum amount of time required for consistently reliable analysis of robot behaviour, and we therefore opt to set W to 29. Figure 4 shows that this value is comfortably in the range that, on average, completely mitigates unresolvable faults in robots.

Sensitivity to proportion of BFV mismatches

In our initial experiments, ρ had almost no correlation with any of the performance criteria. Given the counter-intuitive nature of this observation, we theorised that this may be because the magnitude of contributions of s and W to the performance criteria were obscuring the contributions made by the value of ρ. We then examined the effect ρ had on performance criteria when s and W were held at their optimised values. These additional experiments, which produced Figure 5 and Figure 6, confirmed our suspicions.

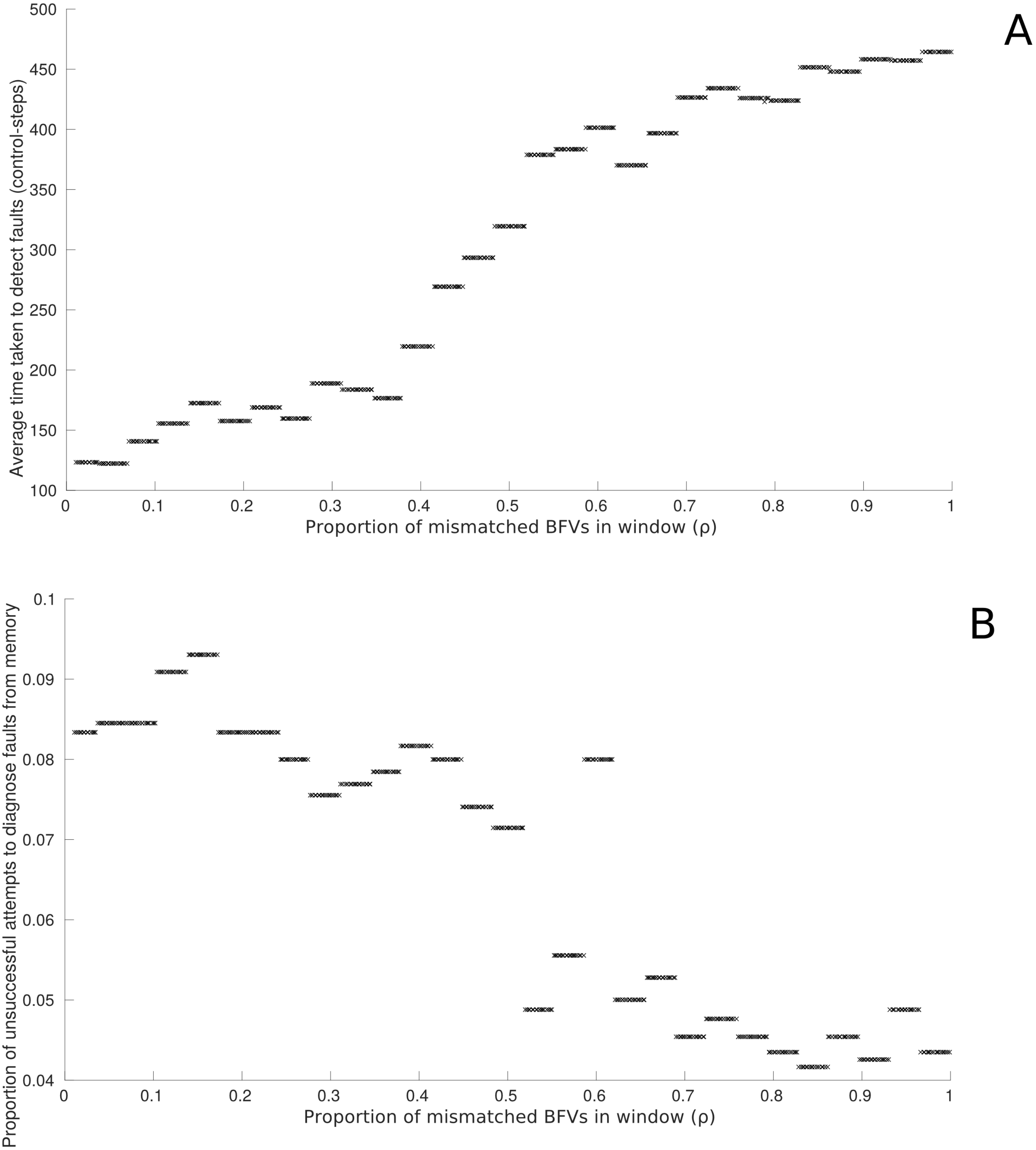

Figure 5. **A**: The median time taken to detect faulty robots against the proportion of mismatched BFVs in the detection window, ρ, and **B**: the median proportion of attempts by the system to diagnose from memory that failed or were not recognised as successful against the proportion of mismatched BFVs in the detection window, ρ

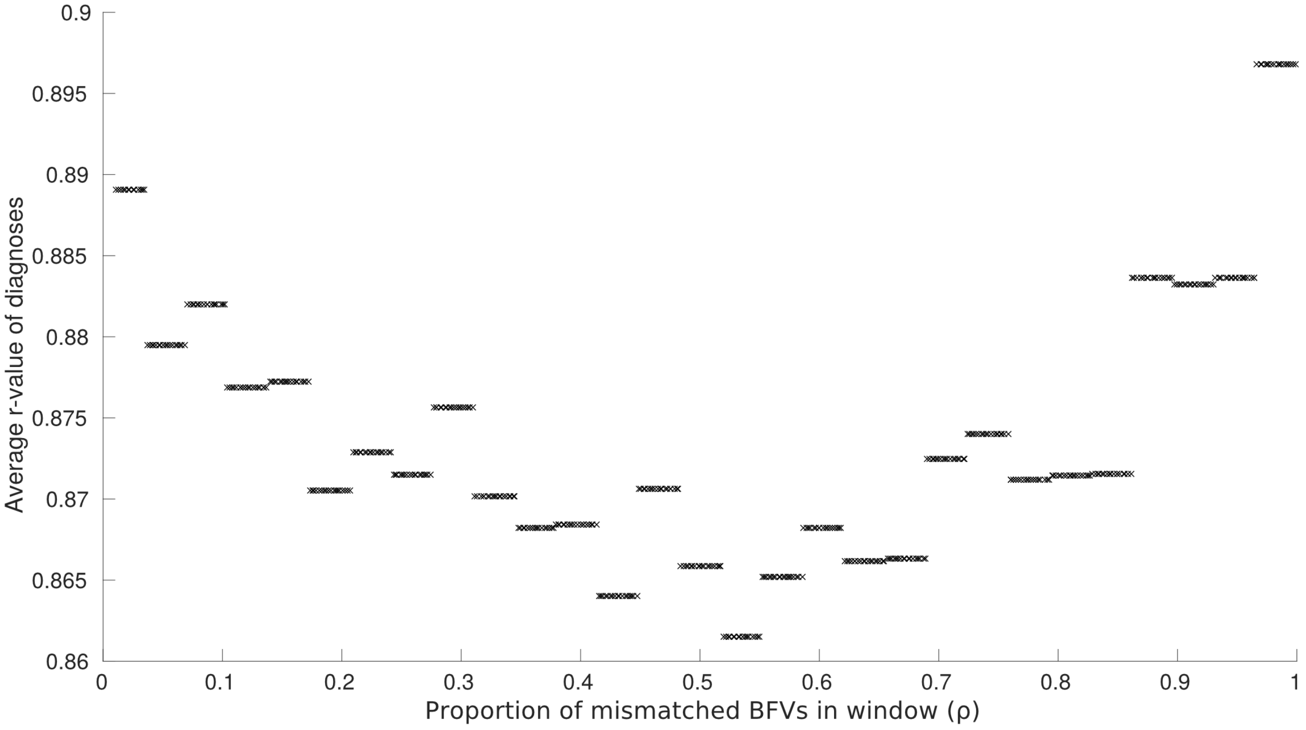

Figure 6. The median r-value of diagnoses against the proportion of mismatched BFVs in the detection window, ρ

The step-like behaviour observed in Figure 5A, Figure 5B and Figure 6 arises from the fact that, as we have set $W = 29$, the value of ρ must increase by approximately 0.035 before it can represent an additional control-step where BFVs were matched or mismatched. As our parameter file generated 500 values between 0.01 and 1, ρ is incremented at a finer granularity than that required to cause any observable change.

There is a positive correlation between the value of ρ and the average time taken to detect faults, P_6, as one would expect, meaning ρ should be minimised in order to minimise the time faulty robots go undetected. Figure 5A shows that this effect is observed over a considerably smaller scale for ρ than W, which would explain why we were not able to observe this correlation when plotting the effects of all s, W, ρ and o on a single graph. This is true of all relations between ρ and system performance.

No strong correlation was observed between the value of ρ and the proportion of faults successfully diagnosed from memory, P_2, however there is a clear negative correlation between increasing values of ρ and the proportion of attempts to diagnose faults from memory that were unsuccessful, P_3 (see Figure 5B). Figure 5B suggests that the optimal value of ρ lies between 0.83 and 0.86 (the precise value of ρ in between this range makes no difference).

Figure 6 shows that the highest average r-values between faults, P_5, are observed as ρ approaches 1 (for $\rho > 0.55$). Interestingly, we observe a quadratic relationship between the value ρ and P_5, with the trough centred in the approximate range $0.5 < \rho < 0.6$ (see Figure 6). Obviously, when ρ is a high value, it necessitates that the BFV must comprise of instances where a fault is explicitly demonstrated in robot behaviour. Instances of each fault category are therefore more likely to be similar, resulting in high r-values between faults. However, even when the value ρ is very low, robots still need to make observations of the faulty robot for W control-steps (this is another contributing factor to the comparatively small effect of ρ compared to other parameters). If the fault type heavily effects robot behaviour, this will be represented in the BFV irrespective

of the value ρ. If the fault type is more tolerable, there is an increased chance the robot will exhibit normal behaviour for a greater proportion of the period W, which will also result in an increased r-value compared to instances where ρ is in a mid-range. This is because instances where faults are more tolerable will necessarily then be observed as a combination of normal and faulty behaviour with a random distribution. However, this effect is relatively insignificant on overall performance, as demonstrated by the small scale of Figure 6. We believe that the reason the relationship between P_5 and ρ is stronger for $\rho > 0.55$ is because, where the value ρ is very low, there is an element of luck present in the BFV representations produced by robots, whereas higher ρ values will produce BFVs that reliably represent the fault present and so produce higher r-values.

The value of ρ was observed to have no effect on the total number of unresolvable faults ,P_4.

Based on our observations, we set $\rho = 0.86$. This value gives us an average detection time of approximately 450 control-steps – an increase of 335 from the theoretic minimum of 115. Given that we demonstrated in O'Keeffe et al. (2017) that the average time taken to run diagnostic tests is approximately 180 control-steps, and that these may have to be run multiple times if our system does not diagnose faults correctly the first time, we consider the compromise on detection time necessitated by choosing a high value for ρ to be worthwhile.

CONFLICT OF INTEREST STATEMENT

The authors declare that the research was conducted in the absence of any commercial or financial relationships that could be construed as a potential conflict of interest.

AUTHOR CONTRIBUTIONS

JO: Performed all experiments and obtained all results described. Principal author of this paper.
DT: Critical revisions.
AGM: Critical revisions.
JT: Critical revisions.

FUNDING

This work was funded by the studentship, awarded by the EPSRC to JO.

DATA AVAILABILITY STATEMENT

The datasets produced and analysed for this study can be found in here: `https://zenodo.org/record/1467221#.W8n7mvn27mE`.

REFERENCES

O'Keeffe, J., Tarapore, D., Millard, A. G., and Timmis, J. (2017). Fault diagnosis in robot swarms: An adaptive online behaviour characterisation approach. In *Computational Intelligence (SSCI), IEEE Symposium Series on* (IEEE), 1–8
